# An Evaluation of a Working Memory Training Scheme in Older Adults

**DOI:** 10.3389/fnagi.2013.00020

**Published:** 2013-05-23

**Authors:** Laura P. McAvinue, Mara Golemme, Marco Castorina, Elisa Tatti, Francesca M. Pigni, Simona Salomone, Sabina Brennan, Ian H. Robertson

**Affiliations:** ^1^Institute of Neuroscience, School of Psychology, Trinity College Dublin, Dublin, Ireland

**Keywords:** working memory, aging, cognitive training, stress, cognitive decline

## Abstract

Working memory is a cognitive process that is particularly vulnerable to decline with age. The current study sought to evaluate the efficacy of a working memory training scheme in improving memory in a group of older adults. A 5-week online training scheme was designed to provide training in the main components of Baddeley’s (2000) working memory model, namely auditory and visuospatial short-term and working memory. A group of older adults aged between 64 and 79 were randomly assigned to a trainee (*n* = 19) or control (*n* = 17) group, with trainees engaging in the adaptive training scheme and controls engaging in a non-adaptive version of the program. Before and after training and at 3- and 6-month follow-up sessions, trainees and controls were asked to complete measures of short-term and working memory, long-term episodic memory, subjective ratings of memory, and attention and achievement of goals set at the beginning of training. The results provided evidence of an expansion of auditory short-term memory span, which was maintained 6 months later, and transfer to long-term episodic memory but no evidence of improvement in working memory capacity *per se*. A serendipitous and intriguing finding of a relationship between time spent training, psychological stress, and training gains provided further insight into individual differences in training gains in older adults.

## Introduction

Baddeley (1992) defined working memory as a “brain system that provides temporary storage and manipulation of the information necessary for such complex cognitive tasks as language comprehension, learning, and reasoning” (p. 556). Working memory has come to be recognized as having a central role to play in general cognition (Engle et al., 1999; Cowan et al., 2005). In youth, the importance of working memory in learning has been demonstrated in studies showing strong associations between children’s working memory capacity and their academic attainments (Gathercole and Pickering, 2000; Gathercole et al., 2003; Jarvis and Gathercole, 2003; Holmes and Adams, 2006). In older adulthood, working memory has been identified as one of the cognitive processes that shows significant decline with age (Park, 2000; Park et al., 2001; Park and Reuter-Lorenz, 2009). The discovery of the importance of working memory in facilitating complex cognition and its vulnerability to decline with age, has led to a recent surge in the development of training programs aimed at enhancing working memory capacity.

A number of reviewers in the area have concluded in favor of the efficacy of working memory training in improving working memory capacity and the extension of its effects to other cognitive processes such as fluid reasoning and attentional control (Klingberg, 2010; Morrison and Chein, 2011). However, a recent fine-grained analysis of the training literature has cast doubt on this conclusion. Having conducted a detailed review of working memory training in relation to children, young adults, and older adults, Shipstead et al. (2012) criticized previous research on a number of grounds, including: inadequate measurement of working memory, either in relation to the use of single tasks as criterion measures or the conflation of working memory with short-term memory; the use of no-contact control groups, which create a host of confounds when used as a comparison groups; and the use, as outcome measures, of subjective reports, which are vulnerable to participant expectations. They concluded that the efficacy of training schemes in improving working memory capacity has yet to be demonstrated.

Other researchers have reached similarly disappointing conclusions regarding the efficacy of working memory training for older adults. A trend has been identified in which older adults improve their performance on the training task or very similar criterion measures but show limited transfer to dissimilar measures of working memory or other cognitive tasks. It has been speculated that reduced cognitive plasticity in older age limits the amount of possible improvement to be gained through training (Morrison and Chein, 2011; Shipstead et al., 2012). For example, Dahlin et al. (2008b) examined the effects of 5 weeks of updating training on a criterion updating task and two transfer tasks (*n*-back and stroop) in a group of young and older adults. They reported significant training gains in both groups but transfer effects in the young group only. A second study conducted with a larger sample and a broader battery of tasks found a similar pattern of results (Dahlin et al., 2008a). Buschkuehl et al. (2008) examined the impact of a mixture of span and complex span training in a sample of participants with a mean age of 80. The trainee group showed significant improvements on the training tasks and near transfer to a similar visual short-term memory task but no improvements on several other transfer tasks of digit span, verbal recall, and visual recall. Li et al. (2008) found that updating training using an *n*-back task led to training gains and improvement on a near transfer *n*-back task but a lack of far transfer to complex span tasks in either young (aged 20–30) or older (aged 70–80) participants. In a group of older adults aged between 60 and 70, Brehmer et al. (2011) reported significant improvements on trained tasks and transfer to short-term memory, working memory, and episodic memory tasks. Their training tasks were largely focused on short-term memory span tasks, however. Examining the effects of complex span training, Richmond et al. (2011) reported significant training effects and near transfer to another complex span task, a far transfer effect to some but not all aspects of verbal learning tested, some evidence of a self-reported improvement in everyday attention but no significant transfer to measures of short-term memory, fluid intelligence, or attention.

In a systematic review of randomized controlled trials conducted to examine the efficacy of cognitive training in improving memory in people in the early stages of Alzheimer’s Disease or Dementia, Clare and Woods (2004) pointed to the lack of evidence for the efficacy of such training. They suggested that cognitive training, involving guided practice on a set of standard tasks, lacked immediate relevance or applicability to the everyday lives of older adults. They advocated the development of a more meaningful, individualized approach, termed Cognitive Rehabilitation, which would involve identifying personally relevant goals and developing strategies to achieve these goals. In a single case study and a randomized controlled trial, Clare et al. (2009, 2010) presented evidence for the efficacy of the approach in improving the activities of daily living in older adults with Mild Cognitive Impairment or in the Early Stages of Alzheimer’s Disease, through the identification and achievement of personal goals.

The purpose of the current study was to examine the efficacy of a working memory training scheme in improving the working memory capacity of a group of older adults (aged 64 to 79). The 5-week computerized training scheme was designed to provide practice in each of the main components of working memory represented in Baddeley’s (2000) model, namely, auditory short-term memory, auditory working memory, visuospatial short-term memory, and visuospatial working memory. In an attempt to combine the Cognitive Training and Cognitive Rehabilitation approaches, participants were also asked to set goals in relation to aspects of daily functioning that they would like to see improve following training. The goals were based on aspects of daily functioning which participants felt were impeded by slips of attention or memory. Trainees were compared to a control group that trained on a non-adaptive version of the program. The two groups were compared pre- and post-training and at 3- and 6-month follow-up sessions on a series of measures of short-term memory, working memory, episodic memory and subjective ratings of attention and memory slips, and goal performance and satisfaction.

## Materials and Methods

### Participants

Fifty-two participants were recruited and randomly allocated to the Trainee or Control Group using a minimization procedure (Altman and Bland, 2005) to balance the two groups in terms of gender and age. Sixteen participants dropped out during the 5-week training period, leaving 19 participants in the Trainee Group and 17 in the Control Group who completed the training and pre and post-training assessments. There were no significant differences between these two groups in terms of age, *t*_34_ = −0.75, *p* = 0.46, gender, *χ^2^*(1) = 0.36, *p* = 0.55, or education level, *χ^2^*(3) = 6.18, *p* = 0.1. A further 10 participants dropped out of the study before all four assessments were conducted. Details of the number, gender, age, and education level of trainees and control participants assessed at each time point are presented in Table 1. The two groups did not differ in terms of IQ, as estimated from performance on the National Adult Reading Test (Nelson, 1982), trainee mean of 120.47 (SD = 4.44) vs. control mean of 118.24 (SD = 8.11), *t*_34_ = 1.04, *p* = 0.31. They did not differ significantly in terms of scores on the Mini-Mental State Examination (Folstein et al., 2010), trainee mean of 27.74 (SD = 2.05) vs. control mean of 28.41 (SD = 1.46), *t*_34_ = −1.13, *p* = 0.27, or on the Hospital Anxiety and Depression Scale (Zigmond and Snaith, 1983), trainee mean of 7.74 (SD = 3.46) vs. control mean of 8.94 (SD = 4.1), *t*_34_ = −0.96, *p* = 0.35.

**Table 1:** **Number, gender, age, and education levels of trainees and controls participating in pre-training, post-training, 3-month follow-up, and 6-month follow-up assessments**.

		Pre and post-training		3-Month follow up		6-Month follow up	
		Trainee	Control	Trainee	Control	Trainee	Control
*n*		19	17	16	14	15	11
Gender	m, f	6, 13	7, 10	6, 10	7, 7	5, 10	5, 6
Age	*M* (SD)	69.89 (4.5)	71.06 (4.8)	70.94 (4.11)	72.14 (4.54)	70.73 (4.17)	72.73 (4.2)
Education	a, b, c, d	1, 2, 10, 6	1, 8, 5, 3	1, 2, 9, 4	1, 5, 5, 3	1, 2, 8, 4	1, 4, 3, 3

*Education: a, Primary School; b, Leaving Certificate; c, Undergraduate; d, Postgraduate*.

### Materials

#### Participant characteristics

##### Mini-mental state examination-2

This is a brief 30 item questionnaire which taps areas such as orientation, memory, attention, and language in order to screen for cognitive impairment (Folstein et al., 2010).

##### The national adult reading test

An estimate of IQ was derived from performance on the NART, which comprises of 50 irregular words, which are read aloud and scored for accuracy (Nelson, 1982).

##### The hospital anxiety and depression scale

The HADS was administered during each assessment session as a self-report measure of current psychological stress (Zigmond and Snaith, 1983).

#### Short-term and working memory

##### Wechsler adult intelligence scale-III

*Digit Span Forwards* was used as a measure of short-term memory. Participants listened to and subsequently repeated a series of numbers read out by the research assistant. Possible scores ranged from 0 to 16. *Digit Span Backwards* was used as a measure of working memory. Participants listened to and subsequently repeated, in reverse order, a series of numbers read out by the research assistant. Possible scores ranged from 0 to 14. *Letter-Number Sequencing* was used as a measure of working memory. Participants listened to a series of letters and numbers read out by the research assistant in random order and subsequently repeated the series, having re-arranged the order so that numbers were presented first, in numerical order, followed by letters, in alphabetical order. Possible scores ranged from 0 to 21 (Wechsler, 1997).

#### Episodic memory

##### Rey auditory verbal learning test

Word lists from the RAVLT were used to assess immediate and delayed memory for words. Different word lists were used for each testing session (i.e., forms AB, Cr-AB, Ge-AB, CD). Word lists consisted of 15 words which were read aloud by the research assistant and recalled verbally by the participant five times to facilitate verbal learning. A distractor word list, also consisting of 15 words, was then read aloud by the research assistant and recalled by the participant. The participant was then asked to recall once more the target word list. The participant’s score on this recall trial served as his/her score for immediate word recall. Delayed recall was tested by asking participants to recall the target word list 20 min later. Possible scores ranged from 0 to 15 (Schmidt, 2004).

##### Rivermead behavioral memory test

Short passages of prose from the first and third editions of the RBMT were used to assess immediate and delayed story recall. Different passages were used for each testing session. The research assistant read the passage aloud to the participant who was then asked to recall (verbally) as much as he/she could of the passage (immediate recall). Delayed story recall was assessed by asking participants to recall the passage once again 20 min later. Possible scores ranged from 0 to 21, with one point being given for each of 21 “ideas” recalled (Wilson et al., 1985; RBMT-3; Wilson et al., 2008).

#### Subjective attention and memory rating

##### Attention-related cognitive errors scale

The ARCES was used as a self-report measure of attention slips and absentmindedness in everyday life. It consists of 12 statements, each of which describes a particular instance of an attentional slip. Participants rate the frequency with which they experience such slips of attention along a scale ranging from 1 (never) to 5 (very often). Possible scores range from 12 to 60, with higher scores representing a higher degree of absentmindedness in everyday life (Carriere et al., 2008).

##### Memory failures scale

Fashioned in the same way as the ARCES, the MFS is a self-report measure of minor memory failures which occur in everyday life. Participants rate the frequency with which they experience memory failures described in a series of 12 statements, which are rated along a scale ranging from 1 (never) to 5 (very often). Possible scores range from 12 to 60, with higher scores representing a higher occurrence of memory failures in everyday life (Carriere et al., 2008).

#### Goals questionnaire

An instrument was developed to facilitate participants in choosing and rating goal activities. The Goals Questionnaire was modeled on the Canadian Occupational Performance Measure (Law et al., 2005), which is an individualized measure designed for use by occupational therapists to detect changes in a client’s self perception of performance of daily living activities over time. During the pre-training assessment session, participants were, first of all, asked to identify from a prepared list, those attentional and memory slips which happen to them from time to time in their daily lives. The list was motivated by examples in the ARCES and MFS questionnaires and participants were also prompted to supply personal examples, if missing from the list. In a second step, participants returned to the list and rated each chosen attentional or memory slip in terms of how troublesome it was in their daily lives, along a scale ranging from 1 (does not trouble me at all) to 10 (extremely troubling). In Step 3, participants were prompted to adopt the five slips which they had rated as being most troublesome in daily life as their goal activities, activities that they would most like to improve as a result of the working memory training. They then rated each of the five goal activities in terms of their current performance of the activity, along a scale ranging from 1 (not able to do it at all) to 10 (able to do it extremely well) and their satisfaction with this performance, along a scale ranging from 1 (not satisfied at all) to 10 (extremely satisfied). An average performance and satisfaction rating was calculated by adding ratings across goals and dividing by the number of goals. During each of the subsequent assessment sessions (post-training, 3-month follow-up, and 6-month follow-up), participants revisited their goal list and supplied current performance and satisfaction ratings. During the post-training assessment session only, participants also indicated the extent to which they felt they had achieved their goals following working memory training, along a scale ranging from 1 (fully achieved) to 10 (not achieved). A single achievement rating was calculated by averaging ratings across goals.

#### Working memory training scheme

The working memory training scheme was designed to provide participants with practice in the components of working memory outlined in Baddeley’s (2000) model, which were auditory short-term and working memory and visuospatial short-term and working memory. It was a computerized program and was run online, with each participant having his/her own login ID and password, which enabled access to the website. The program consisted of a mixture of psycho-education on the nature of working memory and strategies to support its function in everyday life and practice of nine training exercises, which were introduced gradually over the 5 weeks. Participants were asked to practice the training exercises for at least 30 min each day, for 5 days out of each week, for 5 weeks. The training exercises consisted of the following:

*Span tasks* were fashioned after the classic digit span tasks (e.g., Wechsler, 1997).

*Span Numbers* – participants listened to a series of numbers played by the computer and at the end of the sequence, re-entered the numbers either in the same (Span Numbers Normal) or reverse (Span Numbers Reverse) order.
*Span Colors* – participants observed on a colored grid a sequence of flashing colors and reproduced the sequence either in the same (Span Colors Normal) or reverse (Span Colors Reverse) order.

*Focus exercises* were similar to the classic running span tasks (Pollack et al., 1959).

*Focus Faces* – participants observed a series of faces appearing one by one on screen and indicated the last *n* faces observed when the sequence ceased.
*Focus Names* – participants observed a series of names appearing one by one on screen and indicated the last *n* names observed when the sequence ceased.

The *Snap! exercises* were modeled upon the classic *n*-back task (Kirchner, 1958). Participants viewed or listened to a series of stimuli and responded through a key press when two stimuli were repeated in sequence or *n* stimuli apart.

*Faces Snap!* – employed faces as stimuli.
*Spaces Snap!* – involved white squares or “spaces” moving around a black background or “parking lot.”
*Names Snap!* – involved a series of auditory names played by the computer.
*Double Snap!* – was a dual task requiring the participant to respond to visual stimuli (spaces) with one response and auditory stimuli (names) with another.

The *MathsMad task* was modeled upon the classic Paced Auditory Serial Attention Task (PASAT; Crawford et al., 1998). Participants listened to a series of numbers (between 1 and 9) being played by the computer and added the current to the previous digit.

The program was designed to be adaptive, beginning with the easiest level for each participant and presenting levels of increasing difficulty as participants’ performance improved. Participants had access to a results graph for each exercise, which they were encouraged to check regularly in order to follow their own progress on each task. The online arrangement also enabled the researchers to monitor each participant’s participation, performance, and time spent training.

At the end of each week, participants were given information on strategies that could be used to support working memory function in everyday life situations, such as remembering phone numbers and checking change in shops and restaurants. The recommended strategies included chunking, visualization, rounding and estimation, reading techniques, and thinking on one’s feet.

#### Control training

The control group engaged in a non-adaptive version of the training program, which maintained all exercises at the easiest level. A number of exercises (Span Numbers Reverse, Span Colors Reverse, Double Snap!, and MathsMad) were removed from the program entirely as it was felt that even at the easiest level, these exercises taxed working memory. Information on strategies to support working memory function in daily life was also removed from the non-adaptive version of the training program.

## Results

### Effects of working memory training

#### Objective measures

Table 2 presents the mean values and standard deviations obtained by the trainee and control groups on each objective memory measure during each assessment session. A mixed analysis of covariance (ANCOVA), including one between subjects variable, Group (two levels: trainee, control), one within subjects variable, Session (three levels: post-training assessment, 3-month follow-up, 6-month follow-up) and one covariate, pre-training assessment, was run for each memory measure. *Post hoc* one way ANCOVAs were run to examine the effects of Group on each assessment session separately. Table 3 presents the results in relation to the effect of Group for each of these analyses.

**Table 2:** **Means (SDs) for trainees and controls on objective memory measures from pre-training to 6-month follow up**.

		Pre-training	Post-training	Follow up 3 months	Follow up 6 months
**SHORT-TERM AND WORKING MEMORY**					
Digit span forwards	Trainee	11.21 (2.18)	12.32 (1.83)	12.75 (1.48)	12.67 (1.88)
	Control	11.24 (1.25)	10.76 (2.2)	10.5 (1.56)	10.45 (1.63)
Digit span reverse	Trainee	8.05 (2.37)	9.11 (2.05)	9.63 (2.28)	9.67 (1.8)
	Control	7.94 (1.75)	8.29 (2.39)	8.29 (2.3)	8.91 (1.87)
Letter-number sequencing	Trainee	10.53 (2.88)	10.89 (1.94)	12 (2.88)	12.33 (2.5)
	Control	10.71 (1.8)	10.29 (1.57)	11.36 (2.56)	11.55 (2.7)
**EPISODIC MEMORY**					
Word recall immediate	Trainee	9.84 (3.32)	10.84 (2.97)	11.69 (1.96)	11.8 (2.4)
	Control	10.65 (2.74)	9.35 (3.2)	10.86 (3.48)	10.18 (3.76)
Word recall delayed	Trainee	9.79 (3.51)	10.53 (3.13)	11.69 (2.3)	11.67 (2.53)
	Control	10.41 (3.36)	9.06 (3.05)	11 (3.68)	10.45 (3.42)
Story recall immediate	Trainee	10.42 (3.02)	12.87 (2.66)	11.56 (2.84)	12.57 (2.89)
	Control	9.62 (3.69)	11.41 (2.68)	11.25 (3.51)	12.91 (2.96)
Story recall delayed	Trainee	8.66 (2.37)	10.68 (3.04)	10.56 (2.53)	12.04 (2.87)
	Control	8.15 (3.28)	9.29 (3.18)	9.68 (3.91)	11.36 (3.85)

**Table 3:** **Group effects for objective measures: main effect of Group in Mixed Analysis of Covariance (ANCOVA) examining effect of Group across all three post-training assessments, with *Post hoc* One Way ANCOVAs examining effect of Group for each post-training assessment separately**.

	Mixed ANCOVA: effect of group across all three post-training assessments	One Way ANCOVA		
		Post-training assessment	Follow up 3 months	Follow up 6 months
**SHORT-TERM AND WORKING MEMORY**				
Digit span forwards	*F*(1, 23) = 23.65, *p* < 0.001, η^2^ = 0.51	*F*(1, 33) = 10.35, *p* = 0.003, η^2^ = 0.24	*F*(1, 27) = 18.14, *p* < 0.001, η^2^ = 0.4	*F*(1, 23) = 15.86, *p* = 0.001, η^2^ = 0.41
Digit span reverse	*F*(1, 23) = 2.15, *p* = 0.16, η^2^ = 0.09	*F*(1, 33) = 1.75, *p* = 0.2, η^2^ = 0.05	*F*(1, 27) = 3.18, *p* = 0.09, η^2^ = 0.11	*F*(1, 23) = 1.1, *p* = 0.31, η^2^ = 0.05
Letter-number sequencing	*F*(1, 23) = 1.07, *p* = 0.31, η^2^ = 0.04	*F*(1, 33) = 1.81, *p* = 0.19, η^2^ = 0.05	*F*(1, 27) < 1, η^2^ = 0.02	*F*(1, 23) < 1, η^2^ = 0.01
**EPISODIC MEMORY**				
Word recall immediate	*F*(1, 23) = 3.37, *p* = 0.079, η^2^ = 0.13	*F*(1, 33) = 4.68, *p* = 0.04, η^2^ = 0.12	*F*(1, 27) = 2.7, *p* = 0.11, η^2^ = 0.09	*F*(1, 23) = 2.55, *p* = 0.12, η^2^ = 0.1
Word recall delayed	*F*(1, 23) = 2.73, *p* = 0.11, η^2^ = 0.11	*F*(1, 33) = 4.37, *p* = 0.04, η^2^ = 0.12	*F*(1, 27) = 1.18, *p* = 0.29, η^2^ = 0.04	*F*(1, 23) = 1.87, *p* = 0.18, η^2^ = 0.08
Story recall immediate	*F*(1, 23) = 1.08, *p* = 0.31, η^2^ = 0.05	*F*(1, 33) = 2.09, *p* = 0.16, η^2^ = 0.06	*F*(1, 27) < 1, η^2^ = 0.002	*F*(1, 23) < 1, η^2^ = 0.007
Story recall delayed	*F*(1, 22) = 1.71, *p* = 0.2, η^2^ = 0.07	*F*(1, 33) = 1.62, *p* = 0.21, η^2^ = 0.05	*F*(1, 27) < 1, η^2^ = 0.02	*F*(1, 22) < 1, η^2^ = 0.01

**Figure 1:**
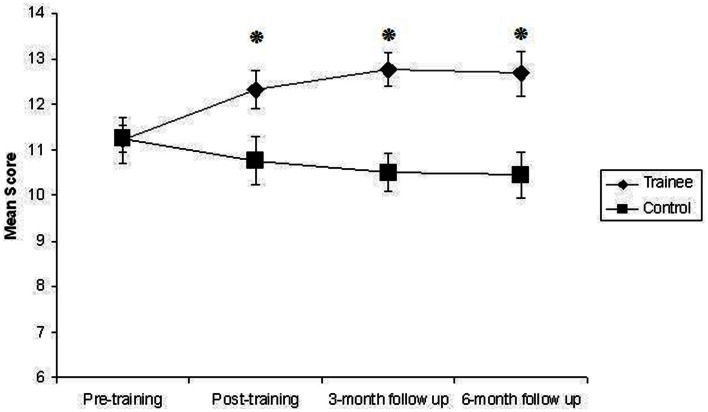
**Mean score for trainees and controls on Digit Span Forwards during pre-training, post-training, 3-month follow-up, and 6-month follow-up assessment sessions**. Error bars represent standard error of the mean. *Indicates statistically significant difference.

**Figure 2:**
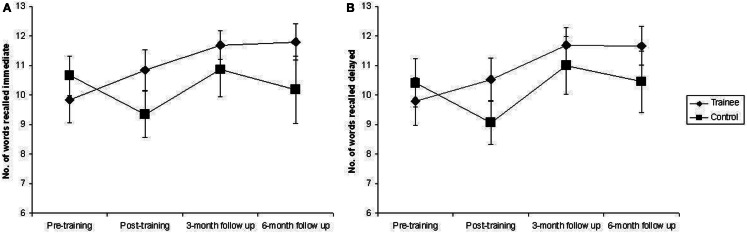
**Mean number of words recalled immediately and after a 20-min delay by trainees and controls during each assessment session**. Error bars represent standard error of the mean. **(A)** Immediate recall. **(B)** Delayed recall.

There was a statistically significant effect of training, with large effect size, η^2^ = 0.51, on Digit Span Forwards, *F*(1, 23) = 23.65, *p* < 0.001. Trainees improved significantly more than controls from the pre-training to the post-training assessment, *F*(1, 33) = 10.35, *p* = 0.003, η^2^ = 0.24, and this improvement was maintained at the 3-month follow-up, *F*(1, 27) = 18.14, *p* < 0.001, η^2^ = 0.4, and at the 6-month follow-up, *F*(1, 23) = 15.86, *p* = 0.001, η^2^ = 0.41, assessments. This improvement is clearly illustrated in Figure 1. The effect of Group in the Mixed ANCOVA for Immediate Word Recall was approaching significance, *F*(1, 23) = 3.37, *p* = 0.079. The *Post hoc* One Way ANCOVAs revealed that trainees improved significantly more than controls from the pre to the post-training assessment, *F*(1, 33) = 4.68, *p* = 0.04, η^2^ = 0.12, but this improvement was not maintained at the 3-month, *F*(1, 27) = 2.7, *p* = 0.11, η^2^ = 0.09, or 6-month, *F*(1, 23) = 2.55, *p* = 0.12, η^2^ = 0.1, follow-up assessments. A similar pattern was evident for Delayed Word Recall. The Mixed ANCOVA was not statistically significant, *F*(1, 23) = 2.73, *p* = 0.11, η^2^ = 0.11, but the *Post hoc* One Way ANCOVAs revealed that trainees improved significantly more than controls from the pre to post-training assessment, *F*(1, 33) = 4.37, *p* = 0.04, η^2^ = 0.12. This improvement was not maintained at the 3-month, *F*(1, 27) = 1.18, *p* = 0.29, η^2^ = 0.04, or 6-month, *F*(1, 23) = 1.87, *p* = 0.18, η^2^ = 0.08, follow-up assessments. Figure 2 displays the pattern of performance of trainees and controls across all assessment sessions on Immediate and Delayed Word Recall. As is clear from Table 3, the effect of training was not statistically significant for Digit Span Reverse, Letter-Number Sequencing, Immediate Story Recall, or Delayed Story Recall.

#### Subjective measures

Table 4 presents the mean values and standard deviations obtained by the trainee and control groups on each subjective memory measure during each assessment session. Table 5 presents the results in relation to the effect of Group for each of the ANCOVAs.

**Table 4:** **Means (SDs) for trainees and controls on subjective memory measures from pre-training to 6-month follow up**.

		Pre-training	Post-training	Follow up 3 months	Follow up 6 months
ARCES	Trainee	30.32 (6.6)	30.32 (6.42)	31.38 (5.99)	30.67 (5.98)
	Control	29.76 (6.03)	28.76 (5.95)	29.07 (5.48)	30.18 (5.81)
MFS	Trainee	30.95 (8.16)	29.26 (7)	30.88 (5.97)	30.47 (6.65)
	Control	29.06 (5.11)	28.12 (4.41)	28.07 (4.34)	28.64 (3.85)
Goal performance	Trainee	4.62 (1.35)	6.26 (1.15)	6.26 (1.37)	6.33 (1.34)
	Control	4.14 (1.12)	5.89 (0.99)	5.99 (1.17)	6.03 (0.92)
Goal satisfaction	Trainee	3.75 (1.63)	6.04 (1.3)	6.09 (1.74)	6.21 (1.42)
	Control	3.70 (1.29)	5.60 (1.01)	6.29 (1.37)	6.26 (1.41)
Goal achievement	Trainee	/	5.24 (1.58)	/	/
	Control	/	5.67 (0.88)	/	/

**Table 5:** **Group effects for subjective measures: main effect of Group in Mixed Analysis of Covariance (ANCOVA) examining effect of Group across all three post-training assessments, with *Post hoc* One Way ANCOVAs examining effect of Group for each post-training assessment separately**.

	Mixed ANCOVA: effect of group across all three post-training assessments	One Way ANCOVA		
		Post-training assessment	Follow up 3 months	Follow up 6 months
ARCES	*F*(1, 23) < 1, η^2^ = 0.02	*F*(1, 33) < 1, η^2^ = 0.02	*F*(1, 27) = 1.87, *p* = 0.18, η^2^ = 0.07	*F*(1, 23) < 1, η^2^ < 0.001
MFS	*F*(1, 23) < 1, η^2^ = 0.04	*F*(1, 33) < 1, η^2^ < 0.001	*F*(1, 27) = 1.64, *p* = 0.21, η^2^ = 0.06	*F*(1, 23) < 1, η^2^ = 0.03
Goals performance	*F*(1, 23) < 1, η^2^ = 0.01	*F*(1, 33) < 1, η^2^ = 0.01	*F*(1, 27) < 1, η^2^ < 0.001	*F*(1, 23) < 1, η^2^ = 0.008
Goals satisfaction	*F*(1, 23) < 1, η^2^ = 0.004	*F*(1, 33) = 1.26, *p* = 0.27, η^2^ = 0.04	*F*(1, 27) < 1, η^2^ = 0.009	*F*(1, 23) < 1, η^2^ = 0.002

The tables clearly show that there was no significant difference between trainees and controls during any post-training assessment session in terms of their ratings of attentional and memory slips (ARCES and MFS) or satisfaction and performance of chosen goals. An independent samples *t*-test revealed that there was no significant difference between the Goal Achievement ratings supplied by trainees (*M* = 5.24, SD = 1.58) and controls (*M* = 5.67, SD = 0.88) during the post-training assessment session, *t*_28.85_ = −1.03, *p* = 0.31. Table 6 presents a list of the top 10 goals selected by participants.

**Table 6:** **Top 10 list of goals: the number of participants including the following goals as part of their goal list**.

Goal category	Number of participants	Specific examples
Improving concentration	34	Improve concentration in general (12)
		Concentrating on what people are saying in conversation (11)
		Concentrating during activities, such as reading (3), games (1), watching television (1)
		Avoid/decrease distraction (6)
Remembering people’s names	30	/
Speedy/quick/immediate recall	20	/
Remembering details	17	Remembering important details (3)
		Details of personal experiences such as places visited (6)
		Details of books read or movies/plays seen (6), prices or names of products in shops (2)
Remembering where I put things	15	/
Task management	14	Improve concentration during multi or dual-tasks (5)
		Task completion (5)
		Remembering for what purpose I entered a room (4)
Planning & remembering to do things	13	Remembering important dates/coming events (6)
		Planning (3)
		Remembering to do tasks (4)
Improving memory capacity	9	Improve retention of information (5)
		Improve short-term memory (4)
Improve memory during games	6	Bridge/cards (6) and Golf (1)
Verbal fluency	4	Recall words or word alternatives (3)
		Express self clearly (1)

**Figure 3:**
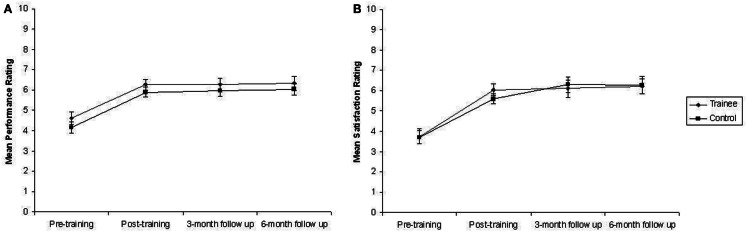
**Mean ratings of performance and satisfaction of goals supplied by trainees and controls during each assessment session**. Error bars represent standard error of the mean. **(A)** Mean performance rating. **(B)** Mean satisfaction rating.

Separate Mixed ANOVAs, with one between subjects variable, Group (two levels: trainee, control) and one within subjects variable, Session (four levels: pre-training, post-training, 3-month follow-up, 6-month follow-up), revealed a significant main effect of Session for ratings of Goal Performance, *F*(3, 72) = 25.66, *p* < 0.001 and Goal Satisfaction, *F*(3, 72) = 31.79, *p* < 0.001, but no Group × Session interaction in either case, *F*(3, 72) < 1. Perusal of the mean values presented in Table 4 and Figure 3 suggests that participants in both groups provided higher Satisfaction and Performance Ratings for Goals in post-training assessments. Indeed, *post hoc* Paired Samples *t*-tests comparing the combined Performance Ratings of both groups across sessions revealed that ratings given during Pre-Training Assessment Session 1 were significantly lower than ratings given during the Post-Training Assessment Session, *t*_35_ = −8.04, *p* < 0.001, the 3-month Follow-Up Session, *t*_29_ = −7.06, *p* < 0.001, and the 6-month Follow-Up Session, *t*_25_ = −5.39, *p* < 0.001, but ratings did not differ significantly between the Post-Training Assessment Sessions, all *p* > 0.05. A similar pattern was found for Satisfaction Ratings, for which ratings during the Pre-Training Assessment Session were significantly lower than those in the Post-Training Assessment Session, *t*_35_ = −7.91, *p* < 0.001, the 3-month Follow-Up Session, *t*_29_ = −8, *p* < 0.001 and the 6-month Follow-Up Session, *t*_25_ = −6.97, *p* < 0.001, whereas ratings did not differ significantly between Post-Training Assessment Sessions, all *p* > 0.05.

### Individual differences in time spent training

Participants were advised to train for 30 min each day but given that the training was completed online in their own homes, they were, of course, masters of their own schedules. This meant that there were individual differences in the amount of time spent training. Overall, trainees spent an average of 14.75 h (SD = 5.25) engaged in the working memory training program. They spent a minimum of 7.88 and a maximum of 26.25 h training. Trainees spent a significantly greater amount of time training than controls, who spent an average of 8.45 h (SD = 3.89) training, *t*_34_ = 4.05, *p* < 0.001.

**Figure 4:**
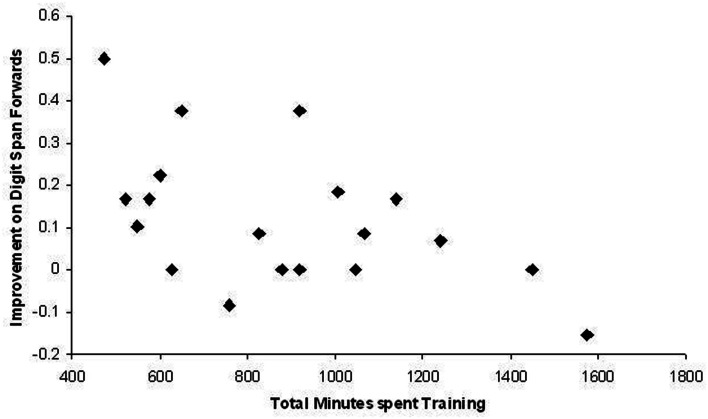
**Scatter plot depicting the relationship between the total number of minutes spent training and proportional improvement on Digit Span Forwards for each trainee**.

In order to examine whether there was a dose-response relationship between time spent training and degree of improvement, correlations were computed between the amount of time spent training by trainees and the proportional improvement during the Post-Training Assessment Session on Digit Span Forwards, Word Recall Immediate, and Word Recall Delayed. Proportional improvement was calculated in the following manner: (Post-Training Assessment Score – Pre-Training Assessment Score)/Pre-Training Assessment Score. The correlations between time spent training and proportional improvement on Word Recall Immediate, *r*_19_ = 0.045, *p* = 0.86, and Word Recall Delayed, *r*_19_ = 0.14, *p* = 0.56, were not statistically significant. The correlation between time spent training and proportional improvement on Digit Span Forwards was statistically significant, *r*_19_ = −0.52, *p* = 0.02, but as is clearly evident in Figure 4, this correlation was in an unexpected direction. It appears that the more time trainees spent training, the less they improved.

Trainees’ data were examined to explore this unexpected finding and significant correlations between time spent training, proportional improvement, and ratings of psychological stress supplied by trainees on the Hospital Anxiety and Depression Scale during the post-training assessment, were uncovered as part of this exploration. There was a significant positive correlation between time spent training and HADS ratings during the post-training assessment, *r*_19_ = 0.62, *p* = 0.005, suggesting the those who had spent more time training reported significantly higher levels of psychological stress during the post-training assessment session. There was a significant negative correlation between HADS score and proportional improvement, *r*_19_ = −0.49, *p* = 0.035, suggesting that those with higher levels of psychological stress during the post-training assessment, improved less. Furthermore, the correlation between time spent training and proportional improvement was reduced to a non-statistically significant level when the HADS score was partialed out, *r*_16_ = −0.32, *p* = 0.19. One possible explanation for this pattern of correlations is that those trainees who put more effort into the training were more anxious or depressed during the post-training assessment and these feelings of psychological stress impeded their performance on the Digit Span Forwards Task during the post-training assessment session.

## Discussion

The current study sought to examine the efficacy of an online working memory training scheme in a group of adults aged between 64 and 79. In comparison to the control group, the trainees showed a significant improvement on a test of auditory short-term memory, Digit Span Forwards, which was maintained at 3- and 6-month follow-up assessment sessions. They also showed a significant improvement in episodic memory on Immediate and Delayed Word Recall Tasks but this improvement was not maintained at follow-up. Trainees did not improve significantly more than controls on auditory working memory (Digit Span Reverse and Letter-Number Sequencing) or in Story Recall episodic memory tasks. There were no significant changes in either group in relation to self-reported memory and attention slips. Both groups gave significantly higher Performance and Satisfaction Ratings for their chosen goal activities during the Post-Training Assessments.

The current findings are in keeping with previous training literature in so far as they indicate a significant improvement in short-term memory but fall short of demonstrating an improvement in working memory, despite the provision of extensive training on tasks modeled upon classic updating and *n*-back tasks. Shipstead et al. (2012) have suggested that an improvement in working memory capacity following cognitive training has yet to be demonstrated and describe how current claims of enhanced working memory capacity can be explained by other aspects of experimental design or measures used.

Previous studies of working memory training in older adults have identified a trend in which older adults show improvements on trained tasks or on tasks similar to those used in training but show limited transfer of gains to tasks dissimilar to the training tasks. Some researchers have speculated that reduced cognitive plasticity precludes the transfer of training gains in older adults (Dahlin et al., 2008a; Morrison and Chein, 2011; Shipstead et al., 2012). The improved short-term memory span found in this study is in keeping with this trend in so far as the Digit Span Forwards measure was very similar to the Span Numbers training task. The maintenance of the training effect is particularly impressive, however, given that a rather small amount of training (around 15 h on average) served to expand span of auditory short-term memory on a rather prolonged basis (up to 6 months). Furthermore, the significant improvement in Immediate and Delayed Word Recall identified during the Post-Training Assessment provides evidence of transfer to episodic memory. Links between short-term and long-term memory have been made (Nee and Jonides, 2008). The expansion of auditory short-term memory span may have facilitated trainees in encoding or retrieval of words during the Word Recall Task (see Cantor and Engle, 1993; Burgess and Hitch, 2005).

The serendipitous finding of the relationship between time spent training, psychological stress, and proportional improvement may also shed some light on lack of improvements in older adults following training. The effect of individual differences on training outcomes has remained largely unexplored (Morrison and Chein, 2011). In this study, a significant negative correlation between time spent training and proportional improvement on the Digit Span Forwards task indicated that those trainees who spent more time training tended to improve less. Further exploration of this curious finding uncovered a significant positive correlation between time spent training and self-report scores on the HADS questionnaire supplied during the Post-Training Assessment, indicating that those who trained more had a tendency to report a higher degree of psychological stress during the Post-Training Assessment. Crucially, the correlation between time spent training and proportional improvement was reduced to a non-statistically significant level when the HADS score was partialed out, prompting the possible explanation that those trainees who put more effort into the training were more anxious or depressed during the Post-Training Assessment and these feelings of psychological stress impeded their performance. The negative impact of stress on memory has been documented (Neupert et al., 2006). It is indeed possible that previous findings of limited training gains or transfer effects identified in older compared to young adults were due to performance-impeding levels of psychological stress rather than limited cognitive plasticity. Certainly, this finding deserves further exploration. At the very least, future research should be cognizant of the possibility of anxiety or depression impeding performance in older participants.

An attempt was made in this study to combine the Cognitive Training and Cognitive Rehabilitation (Clare and Woods, 2004) approaches by asking participants to specify goals or activities they would like to see improve as a result of training. The top 10 goals chosen by trainees and controls were presented in Table 6. The top three goals referred to improving concentration, remembering people’s names, and quick or speedy recall. Unlike the approach used in Cognitive Rehabilitation, participants were not given any strategies aimed specifically at achieving their goals. They were simply told to be aware of their goals and to attempt to apply their training when situations relating to their goals arose. Both trainee and control participants supplied significantly higher ratings for Performance of and Satisfaction with goal activities during the Post-Training Assessment and ratings were maintained at this higher level during the 3- and 6-month follow-up sessions. The similar improvement reported by both trainee and control groups may represent a placebo effect caused by participants’ expectations or may reflect a genuine improvement caused simply by attending to goal activities. Either way, there is no evidence that the cognitive training engaged in by the trainees had any effect on the achievement of their goals.

A significant limitation of this study is its lack of inclusion of a measure of visuospatial short-term or working memory. A considerable proportion of the training was devoted to the visuospatial modality but the inclusion of a corresponding memory measure was neglected. A second limitation relates to the use of a control group that engaged in a non-adaptive version of the training program. Although the use of a non-adaptive training control group has been hailed as the gold standard in this kind of research (Klingberg, 2010), limitations have also been identified in relation to the training experiences received by the two groups. Inevitably, the training given to controls involves much less rigor and they also miss out on feedback on their performance (Shipstead et al., 2012). In this study, controls trained for an average of just under 8½ h while trainees spent just under 15 h training on average. This likely reflects a lower level of engagement in the control participants engendered by very simple and repetitive training tasks. As an alternative to non-adaptive training, it has been suggested that controls engage in an adaptive training scheme that is focused on another cognitive process or activity (Shipstead et al., 2012). Another possibility would be to provide control participants with training designed to expand short-term memory span only while trainees receive training designed to expand working memory capacity in addition to short-term memory span.

Shipstead et al. (2012) have claimed that a true enhancement of working memory capacity following cognitive training has yet to be demonstrated. The working memory training scheme employed in this study was designed to provide practice in the core elements of Baddeley’s (2000) model and training exercises were based upon classic tasks of short-term and working memory, such as span, updating, and *n*-back tasks. Despite this theoretically motivated and comprehensive training package, an increase in working memory capacity was not detected. There was, however, evidence of a rather durable expansion of short-term memory span coupled with transfer of training gains to a long-term episodic memory task, suggesting a degree of cognitive plasticity in this sample of older adults. The unexpected and intriguing relationships uncovered between time spent training, psychological stress, and training gains should stimulate ideas for further avenues of investigation into individual differences in the enhancement of cognition in older adults.

## Conflict of Interest Statement

The authors declare that the research was conducted in the absence of any commercial or financial relationships that could be construed as a potential conflict of interest.
